# Myocardial Infarction and *AGT* p.Thr174Met Polymorphism: A Meta-Analysis of 7657 Subjects

**DOI:** 10.1155/2021/6667934

**Published:** 2021-05-03

**Authors:** Yan-yan Li, Hui Wang, Hao Wang, Yang-yang Zhang

**Affiliations:** ^1^Clinical Research Center, First Affiliated Hospital of Nanjing Medical University, Nanjing 210029, China; ^2^Department of Geriatrics, First Affiliated Hospital of Nanjing Medical University, Nanjing 210029, China; ^3^Department of Cardiology, First Affiliated Hospital of Nanjing Medical University, Nanjing 210029, China; ^4^Department of General Practice, First Affiliated Hospital of Nanjing Medical University, Nanjing 210029, China

## Abstract

**Background:**

It has been suggested that the a*ngiotensinogen* (*AGT*) gene rs4762 (p.Thr174Met) polymorphism might be associated with myocardial infarction (MI) risk, but the study results are still debatable. *Objective and Methods*. In order to explore the relationship between *AGT* p.Thr174Met polymorphism and MI risk, the current meta-analysis involving 7657 subjects from 11 individual studies was conducted.

**Results:**

A significant association between *AGT* p.Thr174Met polymorphism and MI was found under recessive (OR: 2.26, 95% CI: 1.35-3.77, *P* = 0.002), dominant (OR: 1.131, 95% CI: 1.016-1.260, *P* = 0.024), codominant (OR: 2.198, 95% CI: 1.334-3.621, *P* = 0.002), and additive (OR: 1.363, 95% CI: 1.132-1.641, *P* = 0.001) genetic models. In the Asian subgroup, significantly increased MI risk was found under all genetic models (*P* < 0.05). No significant association between *AGT* p.Thr174Met polymorphism and MI was found under all genetic models in the Caucasian subgroup (*P* > 0.05).

**Conclusions:**

*AGT* p.Thr174Met variant might increase MI risk, especially within the Asian population. The Met174 allele of *AGT* p.Thr174Met might confer the risk for MI.

## 1. Introduction

Myocardial infarction (MI) is a serious type of coronary artery disease (CAD) that has high morbidity and mortality rates [1, 2]. MI may be caused by coronary artery critical stenosis or occlusion and myocardial ischemic necrosis based on coronary artery atherosclerosis; however, the pathogenesis has not yet been completely clarified [3, 4]. Risk factors, such as smoking, diabetes mellitus, hypercholesterolemia, gender, and age, are confirmed to increase MI susceptibility. Basic research has shown that individual gene heterogeneity is another risk factor for MI [5]. Hence, MI is a multifactorial disease resulting from heredity and environment factors [6, 7]. In the past few years, many studies that investigated the relationship between gene polymorphisms and MI focused primarily on enzymes and key proteins related to the regulation and metabolism of the cardiovascular system. For example, renin-angiotensin system (RAS), lipid metabolism, coagulation, fibrinolysis, and inflammation system have become hot research spots [8–10]. RAS plays an important role in cardiomyocyte growth regulation, water and electrolyte balance, and blood pressure regulation.

Angiotensinogen (AGT) is the main member of the RAS system. Human AGT is produced in the liver, digested by renin enzyme to produce angiotensin I (Ang I), and then converted to angiotensin II (Ang II) by angiotensin-converting enzyme (ACE). Ang II induces the proliferation and hypertrophy of human vascular smooth muscle cells (VSMCs), increases the expression of platelet-derived growth factor, inhibits fibrinolytic activity, and promotes blood thrombogenesis, low-density lipoprotein (LDL) deposition, oxidation, and endocytosis in the vascular wall [11]. As a salt-sensitive gene, the human *AGT*, located at 1q42-43, is 13 kb in length and contains five exons and four introns. In 1992, Corvol et al. [12] discovered a point mutation substituting thymine (T) for cytosine (C) at position 521 of the exon 2 (3389C>T, rs4762), resulting in methionine (Met) in place of threonine (Thr) at amino acid 174 (p.Thr174Met). The Met174 allele of *AGT* p.Thr174Met might increase the plasma levels of AGT and Ang II, thereby increasing vasoconstriction, VSMC proliferation, and lipid deposition; this allele participates in the pathogenesis of atherosclerosis, hypertension, and MI and increases the MI risk [11, 13–15].

Although studies on the relationship between *AGT* p.Thr174Met polymorphism and MI were widely performed, their results varied widely. In 2006, Ning et al. [16] found that *AGT* Met174 allele increased MI risk in the Chinese population. In 2008, Xie et al. [17] also found that the MM genotype and M allele of *AGT* p.Thr174Met might be a risk factor for MI in an Uigur ethnic population in Xinjiang of China. By contrast, in 1995, Tiret et al. [18] reported that the *AGT* locus had no significant impact on the risk of nonfatal MI in the French population.

This meta-analysis including 3944 patients with MI and 3713 controls was conducted to confirm the relationship between *AGT* p.Thr174Met polymorphism and MI.

## 2. Publication Search and Inclusion Criteria

The terms “myocardial infarction”, “angiotensinogen”, and “polymorphism” were used to search the China Biological Medicine Database China National Knowledge Infrastructure, Embase, PubMed, and Web of Science. Studies were retrieved within the publication year ranging from 1995 to 2018 (the last research updated on April 20, 2021).

The inclusion criteria for the studies were as follows: (a) evaluation of the *AGT* p.Thr174Met polymorphism and MI and (b) diagnosis of MI in accordance with the diagnosis criteria promulgated by the World Health Organization in 1979 or the global universal definition of MI by the European Society of Cardiology, American College of Cardiology Foundation, American Heart Association, and World Heart Federation in 2012. MI was diagnosed if the patient had a conclusive positive history of MI or presented with ST elevation or non-ST elevation.

### 2.1. Data Extraction

Data were obtained according to a standard protocol. Repeated literature, studies that did not meet the inclusion criteria, and studies with limited data were removed. Overlapping portions of data were used only once. The Newcastle-Ottawa Scale (NOS) was used to evaluate the quality of all eligible studies. The abstracted data were composed of the following items: the first author's name, publication year, region, number of genotypes, genotyping, study design, matching criteria, total number of cases and controls, *P* value for Hardy-Weinberg equilibrium (HWE), and NOS score.

### 2.2. Statistical Analysis

All of the statistical analysis would be performed by using the STATA 12.0 software (StataCorp, College Station, TX) and review manager 5.0, and *P* value was set at <0.05 for most of the statistical tests.

In order to assess the association between *AGT* p.Thr174Met polymorphism and MI, the pooled odds ratio (OR) corresponding to 95% confidence interval (CI) was used. In the present meta-analysis, four genetic models as the recessive (MetMet vs. ThrMet+ThrThr, MM vs. TT+TM), dominant (MetMet+ThrMet vs. ThrThr, MM+TM vs. TT), codominant (MetMet vs. ThrThr, MM vs. TT), and additive genetic models (Met vs. Thr, M vs. T) were used. The chi-square test was used to evaluate the HWE of the genotype distribution.

In order to calculate the heterogeneity between studies, the chi-square-based *Q*-test was used with significance set at *P* < 0.10 [19]. The inconsistency index *I*^2^ was calculated to assess the heterogeneity variation. The pooled OR was determined using *Z* test, and the random-effects model was applied when there was heterogeneity among the studies (DerSimonian and Laird method) [20]. Otherwise, the fixed-effects model was used (Mantel-Haenszel method) [21].

The potential publication bias would be estimated by using a funnel plot. The funnel plot asymmetry would be assessed by using Egger's linear regression test on the natural logarithm scale of the OR [22].

## 3. Results

### 3.1. Studies and Populations

Of the 20 papers produced by our initial search of literature, 11 studies met the inclusion criteria. Of the 9 dropped studies, four papers were double publications, two papers were reviews, and three papers were not associated with the *AGT* p.Thr174Met polymorphism. No study was excluded for the reason of deviating from HWE.

Total data were extracted from 3944 patients with MI and 3713 controls (Table 1) [16–18, 23–30]. Patients were from China, Russia, Germany, Austria, United Kingdom, Poland, Mexico, United Arab Emirates, and France. Two ethnicities present were Asian and Caucasian.

The NOS scores in all of the included studies were no less than 6 stars, which implied that all of the eligible studies were of high quality.

### 3.2. Pooled Analyses

**Figure 1:**
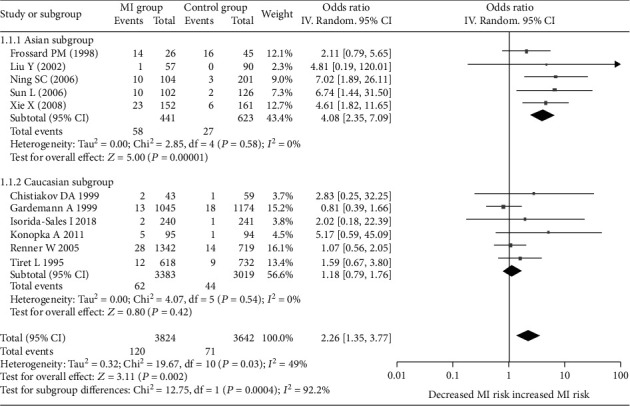
Forest plot of *AGT* p.Thr174Met polymorphism associated with myocardial infarction stratified by ethnicity using a recessive genetic model (MM vs. TM+TT).

**Figure 2:**
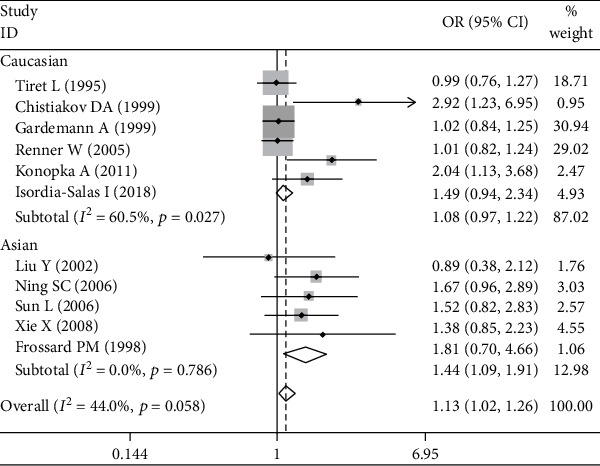
Forest plot of *AGT* p.Thr174Met polymorphism associated with myocardial infarction stratified by ethnicity using a dominant genetic model (TM+MM vs. TT).

**Figure 3:**
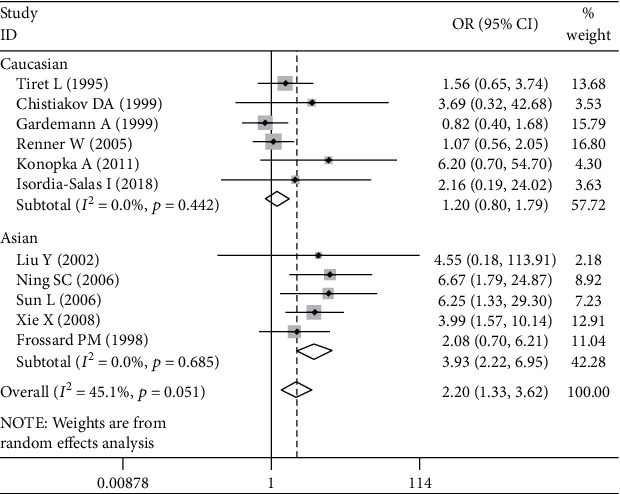
Forest plot of *AGT* p.Thr174Met polymorphism associated with myocardial infarction stratified by ethnicity using a codominant genetic model (MM vs. TT).

**Figure 4:**
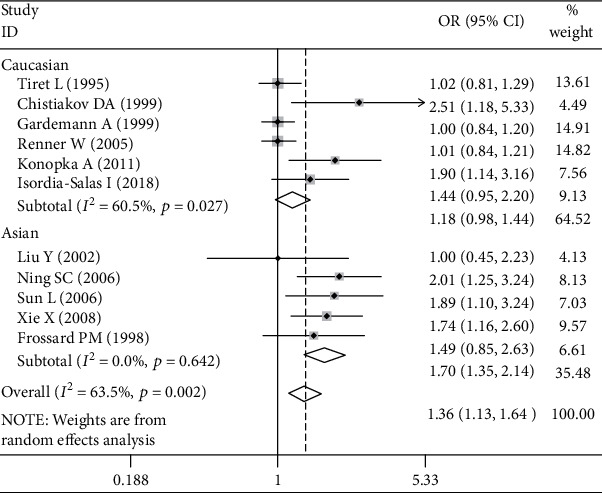
Forest plot of *AGT* p.Thr174Met polymorphism associated with myocardial infarction stratified by ethnicity using an additive genetic model (M vs. T).

Significant associations between *AGT* p.Thr174Met polymorphism and MI were found for recessive (OR: 2.26, 95% CI: 1.35-3.77, *P* = 0.002), dominant (OR: 1.131, 95% CI: 1.016-1.260, *P* = 0.024), codominant (OR: 2.198, 95% CI: 1.334-3.621, *P* = 0.002), and additive (OR: 1.363, 95% CI: 1.132-1.641, *P* = 0.001) genetic models (Table 2, Figures 1–4).

Further association analyses in Asian and Caucasian subgroups were performed. Significant heterogeneity was found in the overall population for all genetic models (*P*_heterogeneity_ < 0.10), but no heterogeneity existed in the Asian subgroup for all genetic models (*P*_heterogeneity_ > 0.10) (Table 2, Figures 1–4). In the Caucasian subgroup, significant heterogeneity was also found for dominant and additive genetic models (*P*_heterogeneity_ < 0.10) (Table 2, Figures 2 and 4), and no significant heterogeneity existed for recessive or codominant genetic models (*P*_heterogeneity_ > 0.10) (Table 2, Figures 1 and 3). Hence, ethnicity was the main heterogeneity source in the current meta-analysis.

In the Asian subgroup analysis, *AGT* p.Thr174Met polymorphism significantly increased MI risk for recessive (OR: 4.08, 95% CI: 2.35-7.09, *P* = 5.73 × 10^−7^, *P*_heterogeneity_ = 0.58), dominant (OR: 1.444, 95% CI: 1.092-1.910, *P* = 0.01, *P*_heterogeneity_ = 0.786), codominant (OR: 3.928, 95% CI: 2.222-6.945, *P* = 2.60 × 10^−6^, *P*_heterogeneity_ = 0.685), and additive (OR: 1.701, 95% CI: 1.350-2.143, *P* = 6.48 × 10^−6^, *P*_heterogeneity_ = 0.642) genetic models (Table 2, Figures 1–4). However, no significant association was found between *AGT* p.Thr174Met polymorphism and MI in the Caucasian subgroup for recessive (OR: 1.18, 95% CI: 0.79-1.76, *P* = 0.42, *P*_heterogeneity_ = 0.54), dominant (OR: 1.085, 95% CI: 0.966-1.219, *P* = 0.171, *P*_heterogeneity_ = 0.027), codominant (OR: 1.195, 95% CI: 0.799-1.788, *P* = 0.386, *P*_heterogeneity_ = 0.442), or additive (OR: 1.185, 95% CI: 0.976-1.438, *P* = 0.087, *P*_heterogeneity_ = 0.027) genetic models (Table 2, Figures 1–4).

**Figure 5:**
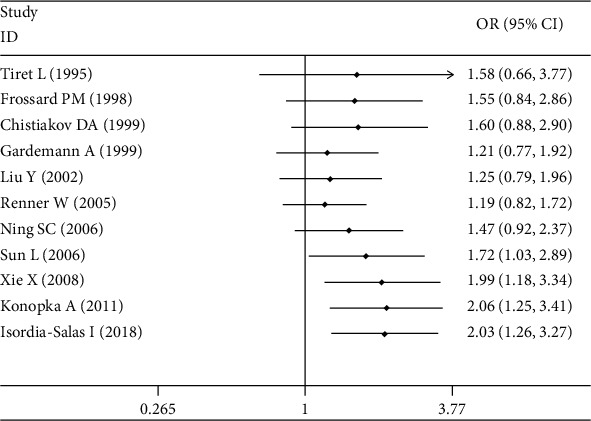
Forest plot of cumulative analysis over time of *AGT* p.Thr174Met polymorphism associated with myocardial infarction using a recessive genetic model (MM vs. TM+TT).

A cumulative analysis over time was performed using the recessive genetic model (Figure 5). No significant association was found between *AGT* p.Thr174Met polymorphism and MI before 2006 (OR: 1.47, 95% CI: 0.92-2.37, *P* = 0.071). In 2006, after the study by Sun et al. was added in the current meta-analysis, a significant association between them was detected (OR: 1.72, 95% CI: 1.03-2.89, *P* = 0.039).

### 3.3. Bias Diagnostics

**Figure 6:**
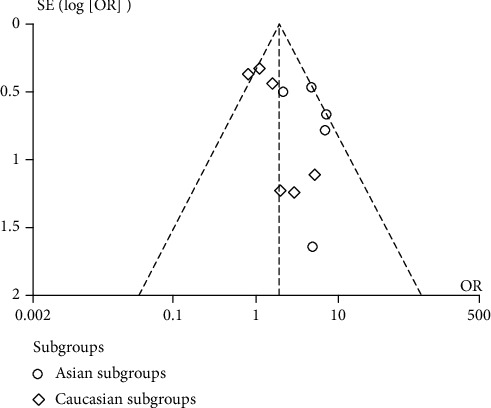
Funnel plot for studies of *AGT* p.Thr174Met polymorphism associated with myocardial infarction using a recessive genetic model (MM vs. TM+TT). OR: odds ratio; SE: standard error.

**Figure 7:**
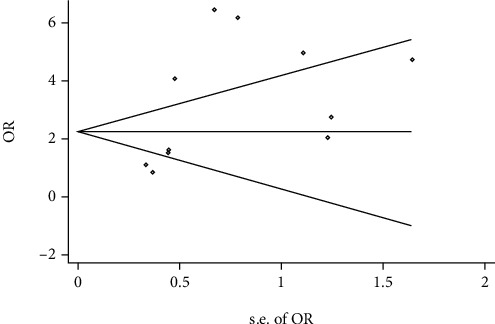
Begg's funnel plot for studies of *AGT* p.Thr174Met polymorphism associated with myocardial infarction using a recessive genetic model (MM vs. TM+TT). OR: odds ratio; SE: standard error.

Funnel plot and Egger's test were used to assess the publication bias of the individual studies. No visual publication bias was found according to the funnel plot of the overall studies using a recessive genetic model (Figure 6). Further, no significant difference was detected in Egger's test, suggesting lack of publication bias in this meta-analysis by using the recessive genetic model (*T* = 2.16, *P* = 0.059; Figure 7).

## 4. Discussion

In this meta-analysis, the controls comprised hospitalized individuals who matched the case group by age, sex, BMI, or ethnicity. The controls were not diagnosed with CAD by coronary angiography and had no history of cardiovascular disease in their families. Case-control study design was used in all of the included studies. The abstracted data quality was high because the NOS score in all of the included studies, including manuscript selection, comparability, and exposure, was no less than 6 stars.

In the entire sample population, a significant association was found between *AGT* p.Thr174Met polymorphism and MI for recessive (OR: 2.26), dominant (OR: 1.131), codominant (OR: 2.198), and additive (OR: 1.363) genetic models.

The different results in the two subgroups suggested that ethnicity might influence the association between *AGT* p.Thr174Met polymorphism and MI. Furthermore, the allelic frequencies between these ethnic groups and the differences in the matching criteria could also influence the results. In addition, the negative results in the Caucasian population were possibly influenced by the small number of included studies. Only six studies were included in the Caucasian subgroup, indicating that more studies should be conducted in the future to verify the result within this population.

The cumulative analysis over time also showed that the significant association between MI and *AGT* p.Thr174Met variant was found after 2006. It was suggested that the significant association between them was confirmed after 2006 which might be related with more individual included studies. The bias diagnostics showed that no publication bias was detected which suggested that no obvious systemic error existed in the current meta-analysis.

MI is a multifactorial disease that varies among race, ethnicity, and geographic region. Different morbidity rates were found among different races and ethnicities. Although the *AGT* p.Thr174Met mutation is not in the range of conserved sequences of the AGT protein core serpin domain [31, 32], this mutation involves the conversion between hydrophobic Met and hydrophilic Thr, and in the 3D structure of AGT protein, the adjacent Thr at 174 is Thr206. Therefore, it is speculated that the result of this mutation may be the interaction between amino acids with different polarities and the conformational change that increases the activity of AGT in RAS. Thus, it affects the activation or inactivation of the corresponding components in the coagulation and fibrinolysis system and promotes the formation of MI [33].

In 2012, Xu et al. [34] performed a meta-analysis on the association of *AGT* p.Thr174Met polymorphism and CAD risk and found no significant overall association (OR: 1.07, CI: 0.93-1.22) in the Asian and Caucasian population. Their meta-analysis included 16 individual studies. CAD includes angina pectoris, MI, silent myocardial ischemia, ischemic cardiomyopathy, and sudden death. The different results between all types of CAD, single MI risk, and *AGT* p.Thr174Met polymorphism were probably associated with different genetic variants among the five different types of CAD diseases.

A further meta-analysis found association of the *AGT* p.Thr174Met polymorphism with MI risk [35] in both Caucasian and Asian populations, but only six individual studies were included. Compared with the previously published meta-analyses on the topic, higher number of individual studies was included in the present work. The present meta-analysis confirmed the significant association between them again by analyzing more studies.

This meta-analysis has some limitations. The sample size is relatively small, and large-scale research on the relationship between MI and *AGT* p.Thr174Met polymorphism remains insufficient. Other *AGT* genetic polymorphisms such as -217G>A (rs5049), -532C>T (rs5046), 12775G>A (rs943580), -6T>C (rs5051), 4072C>T orMet235Thr (rs699), 11535C>A (rs7079), and 6309C>T (rs2493132) might also influence the AGT activity. Additionally, environmental factors, such as race, region, smoking status, hyperlipidemia, and diabetes mellitus, were not accounted for in this study.

## 5. Conclusions

*AGT* p.Thr174Met variant increases MI risk, especially in the Asian population. This finding needs to be further confirmed by further research in the future.

## Figures and Tables

**Table 1:** Characteristics of the selected studies of the association between *AGT* p.Thr174Met polymorphism and myocardial infarction.

Author	Year	Region	Ethnicity	MI			Control			Minor allele frequency		Genotyping	Matching criteria	Sample size (MI/control)	*P* value for HWE	NOS score (stars)
				TT	TM	MM	TT	TM	MM	MI	Control					
Tiret et al. [18]	1995	UK, France	Caucasian	493	125	12	578	154	9	0.118	0.116	AS-PCR	BMI, ethnicity	630/741	0.73	6
Frossard et al. [28]	1998	UAE	Asian	8	18	14	19	26	16	0.1	0.11	PCR-RFLP	Age, sex, BMI, ethnicity	40/61	0.26	6
Chistiakov et al. [23]	1999	Russia	Caucasian	26	17	2	48	11	1	0.322	0.108	PCR-RFLP	Ethnicity	45/60	0.69	6
Gardemann et al. [24]	1999	Germany	Caucasian	817	228	13	925	249	18	0.120	0.120	PCR	Age, ethnicity	1058/1192	0.79	6
Liu et al. [25]	2002	China	Asian	48	9	1	73	17	0	0.095	0.094	PCR	Sex, ethnicity	58/90	0.32	6
Renner et al. [26]	2005	Austria	Caucasian	1017	325	28	545	174	14	0.139	0.138	PCR	BMI, ethnicity	1370/733	0.98	6
Ning et al. [16]	2006	China	Asian	84	20	10	168	33	3	0.175	0.096	PCR-RFLP	Ethnicity	114/204	0.36	6
Sun et al. [27]	2006	China	Asian	84	18	10	105	21	2	0.170	0.098	PCR	Age, sex, BMI, ethnicity	112/128	0.43	6
Xie et al. [17]	2008	China	Asian	122	30	23	127	34	6	0.217	0.153	PCR-RFLP	Age, sex, ethnicity	175/167	0.06	6
Konopka et al. [29]	2011	Poland	Caucasians	54	41	5	67	27	1	0.255	0.153	PCR-RFLP	Sex, ethnicity	100/95	0.34	6
Isordia-Salas et al. [30]	2018	Mexico	Caucasians	187	53	2	202	39	1	0.118	0.085	PCR-RFLP	Age, sex, BMI, ethnicity	242/242	0.54	6

Case-control study design was used in all studies. AS-PCR: allele-specific PCR; BMI: body mass index; MI: myocardial infarction; PCR: polymerase chain reaction; RFLP: restriction fragment length polymorphism; TT: ThrThr; TM: ThrMet; MM: MetMet; UAE: United Arab Emirates; UK: United Kingdom.

**Table 2:** Summary results of meta-analysis on association between *AGT* p.Thr174Met polymorphism and myocardial infarction.

Genetic model	Pooled OR (95% CI)	*Z* value	*P* value	Studies	Sample size		*P*_heterogeneity_ (*I*^2^%)
					MI	Control	
*Recessive*							
Overall group	2.26 (1.35-3.77)	3.11	0.002^∗^	11	3944	3713	0.03^∗∗^ (49%)
Asian subgroup	4.08 (2.35-7.09)	5.00	5.73 × 10^−7∗^	5	499	650	0.58 (0%)
Caucasian subgroup	1.18 (0.79-1.76)	0.80	0.42	6	3445	3063	0.54 (0%)
*Dominant*							
Overall group	1.131 (1.016-1.260)	2.25	0.024^∗^	11	3944	3713	0.058^∗^ (44.0%)
Asian subgroup	1.444 (1.092-1.910)	2.58	0.010^∗^	5	499	650	0.786 (0%)
Caucasian subgroup	1.085 (0.966-1.219)	0.96	0.338	6	3445	3063	0.027^∗∗^ (60.5%)
*Codominant*							
Overall group	2.198 (1.334-3.621)	3.09	0.002^∗^	11	3944	3713	0.051^∗∗^ (45.1%)
Asian subgroup	3.928 (2.222-6.945)	4.70	2.60 × 10^−6∗^	5	499	650	0.685 (0%)
Caucasian subgroup	1.195 (0.799-1.788)	0.87	0.386	6	3445	3063	0.442 (0%)
*Additive*							
Overall group	1.363 (1.132-1.641)	3.26	0.001^∗^	11	3944	3713	0.002^∗∗^ (63.5%)
Asian subgroup	1.701 (1.350-2.143)	4.51	6.48 × 10^−6∗^	5	499	650	0.642 (0%)
Caucasian subgroup	1.185 (0.976-1.438)	1.71	0.087	6	3445	3063	0.027^∗∗^ (60.5%)

^∗^ *P* < 0.05, ^∗∗^*P* < 0.10; CI: confidence interval; OR: odds ratio; MI: myocardial infarction; recessive genetic model: MM vs. TM+TT; dominant genetic model: TM+MM vs. TT; codominant genetic model: MM vs. TT; additive genetic model: total M vs. total T.

## Data Availability

The data used to support the findings of this study are available from the corresponding author upon request.
